# Cold spells and ischaemic sudden cardiac death: effect modification by prior diagnosis of ischaemic heart disease and cardioprotective medication

**DOI:** 10.1038/srep41060

**Published:** 2017-01-20

**Authors:** Niilo R. I. Ryti, Elina M. S. Mäkikyrö, Harri Antikainen, M. Juhani Junttila, Eeva Hookana, Tiina M. Ikäheimo, Marja-Leena Kortelainen, Heikki V. Huikuri, Jouni J. K. Jaakkola

**Affiliations:** 1 Center for Environmental and Respiratory Health Research (CERH), Faculty of Medicine, University of Oulu, PO Box 5000, FI-90014, Finland; 2 Medical Research Center Oulu, Oulu University Hospital and University of Oulu, Oulu, Finland; 3 Department of Geography, University of Oulu, Oulu, Finland; 4 Division of Cardiology, Department of Internal Medicine, University of Oulu, Finland; 5 Department of Forensic Medicine, University of Oulu, Oulu, Finland

## Abstract

Sudden cardiac death (SCD) is the leading cause of death. The current paradigm in SCD requires the presence of an abnormal myocardial substrate and an internal or external transient factor that triggers cardiac arrest. Based on prior mechanistic evidence, we hypothesized that an unusually cold weather event (a cold spell) could act as an external factor triggering SCD. We tested potential effect modification of prior diagnoses and select pharmacological agents disrupting pathological pathways between cold exposure and death. The home coordinates of 2572 autopsy-verified cases of ischaemic SCD aged ≥35 in the Province of Oulu, Finland, were linked to 51 years of home-specific weather data. Based on conditional logistic regression, an increased risk of ischaemic SCD associated with a cold spell preceding death (OR 1.49; 95% CI: 1.06–2.09). Cases without a prior diagnosis of ischaemic heart disease seemed more susceptible to the effects of cold spells (OR 1.70; 95% CI: 1.13–2.56) than cases who had been diagnosed during lifetime (OR 1.14; 95% CI: 0.61–2.10). The use of aspirin, β-blockers, and/or nitrates, independently and in combinations decreased the risk of ischaemic SCD during cold spells. The findings open up new lines of research in mitigating the adverse health effects of weather.

Sudden cardiac death (SCD) is the leading cause of death, representing 50–60% of all cardiovascular deaths^1^,^2^. The current paradigm in SCD requires the presence of an abnormal myocardial substrate, such as coronary heart disease or a genetic disorder, and a transient external or internal factor that triggers cardiac arrest^1^,^3^. Little is known about weather as a potential determinant of SCD^4^,^5^,^6^.

There is substantial evidence that cardiovascular mortality is associated with the ambient temperature of the day or preceding days of the incident^7^,^8^. In addition, prolonged periods of exceptionally cold weather, denoted as cold spells, increase mortality in patterns that are less understood. According to our recent systematic review and meta-analysis, cold spells are associated with approximately 11% increase in cardiovascular mortality rates around the world^9^. The mode of death in these studies is not known. Despite the suggestive epidemiologic evidence and a solid physiological basis discussed later in this paper, there is only a handful of studies on the potential association between cold weather and SCD^4^,^5^,^6^,^10^,^11^, and no studies on the association between cold spells and SCD^9^.

The rationale for our study is that a cold spell, being a transient, sudden, intense weather phenomenon with known effects on the cardiovascular system, could act as a potential trigger of SCD. Establishing an association between cold spells and SCD and the further identification of factors affecting susceptibility would open up previously unexplored possibilities in SCD prevention. A question of practical interest is whether the timely recognition of ischaemic heart disease (IHD) could protect the patient from a later ischaemic SCD during cold weather, and why. Such protection might be accounted to changes in behavior and medication, for example.

The aim of our study was to test the following *a priori* hypotheses: (a) the risk of ischaemic SCD is increased in relation to cold spells; (b) among the patients suffering ischaemic SCD, the relation between cold spells and SCD is stronger among those without a prior diagnosis of IHD compared to those with a prior diagnosis; (c) the relation between cold spells and SCD is weaker among patients using aspirin, β-blockers, and/or nitrates compared to those without this medication. The hypotheses were based on previous mechanistic evidence illustrated in Fig. 1.

## Results

### Characteristics of the study population

Out of the 2572 cases of ischaemic SCD in our study population, 823 (32%) had a prior diagnosis of IHD and 1749 (68%) did not. More specifically, 661 cases (26%) had a prior diagnosis of coronary artery disease (CAD), 264 (11%) had survived a previous acute myocardial infarction (AMI), and 225 (9%) had a prior diagnosis of angina pectoris (AP). Out of the 823 cases with a previous diagnosis of IHD, 44% had been on aspirin, 59% had been on a β-blocker, and 65% had been on a nitrate therapy. Characteristics of the study population are shown in Table 1. Distributions of temperatures and exposure parameters are shown in Table 2.

### Effect modification by age and sex

Based on conditional logistic regression modeling, cold spells were associated with an increased risk of SCD (OR 1.49; 95% CI: 1.06–12.09). The effect estimate was greater for cases aged 35–64 (OR 2.45; 95% CI 0.91–6.64) than for cases aged ≥65 (OR 0.77; 95% CI: 0.34–1.73) in the diagnosed. Men seemed to be more susceptible (OR 1.23; 95% CI: 0.63–2.42) than women in this group (OR 0.78; 95% CI: 0.17–3.63). The effect estimate was greater for cases aged ≥65 (OR 1.99; 95% CI: 1.14–3.45) than for cases aged 35–64 (OR 1.42; 95% CI: 0.77–2.62) in the undiagnosed. Women seemed to be more susceptible (OR 2.35; 95% CI: 0.98–5.66) than men in this group (OR 1.56; 95% CI: 0.98–2.48) (Table 3).

### Effect modification by prior diagnoses

Cases without a prior diagnosis of IHD were more susceptible to the effects of cold spells (OR 1.70; 95% CI: 1.13–2.56) than cases who had been diagnosed during their lifetime (OR 1.14; 95% CI: 0.61–2.10) (Table 3). Similar direction of effect modification was observed for each individual diagnosis of CAD, AMI, and AP (Table 4).

### Effect modification by the use of medications

The effect estimate was greater among cases not using aspirin (OR 1.90; 95% CI: 0.77–4.70) than among those using aspirin (OR 1.02; 95% CI: 0.34–3.08) (Table 5). The effect estimate was greater among cases without nitrate therapy (OR 3.95; 95% CI: 1.52–10.25) than among those who used nitrates (OR 0.52; 95% CI: 0.15–1.74). The effect estimate was greater among cases without β-blockers (OR 1.61; 95% CI: 0.63–4.11) than among those who used β-blockers (OR 1.29; 95% CI: 0.46–3.60). We analyzed the effect modification of β-blockers also in those without a prior diagnosis of IHD. To minimize confounding by indication, we only included cases using β-blockers to treat hypertension in this analysis. Similar direction of effect modification was observed, with an increased risk among the non-users (n = 1009) (OR 1.74; 95% CI: 1.01–2.99) but not among the users of β-blockers (n = 159) (OR 0.95; 95% CI: 0.26–3.41). A declining pattern was observed in the effect estimates between those using none of the studied medications (n = 76) (OR 4.23; 95% CI: 1.06–16.94), those using any of the studied medications (n = 435) (OR 1.05; 95% CI: 0.45–2.42), and those using all of the studied medications (n = 113) (OR 0.51; 95% CI: 0.06–4.22) (Table 5).

## Discussion

There is substantial previous evidence on the association between cold weather and mortality^7^,^8^,^12^, which provides a general framework and rationale for this study. Whether or not and how much SCD might contribute to these effect estimates in unknown and of little practical interest, as outcome-specific evidence is needed for predicting and mitigating SCD^13^. There is compelling evidence on the association between cold weather and acute myocardial infarction, but less is known on the mode of death^14^,^15^. Acute myocardial infarction and sudden cardiac death seem not to respond to mitigation efforts in unison^13^,^16^,^17^,^18^. For this reason, specific studies on the risk factors and mechanisms of SCD are particularly warranted^13^. To our knowledge, there are no previous studies on the association between cold spells and SCD^9^. We showed in an autopsy-verified setting and applying specific personal exposure assessment that there was a positive association between cold spells and ischaemic SCD. Cases without a previously diagnosed IHD seemed to be more susceptible to the effects of cold spells than cases who had been diagnosed during their lifetime. This finding was supported by the same direction of effect modification observed for each diagnosis of CAD, AP, and AMI. Aspirin, β-blockers, and nitrates independently and in combinations seemed to weaken the positive associations observed between cold spells and ischaemic SCD.

There were differences in effect modification by age. SCD is known to commonly occur in the prime of life^1^. This was also observed in our study where those in the prime of life (35–64 years of age) were at the greatest risk during cold spells based on the overall analyses and the stratified analyses for the previously diagnosed. Taken that a clinically manifested disease at a young age might be more severe than a disease that has remained subclinical until older age, this finding is sensible. However, we did not have information on the date when the prior diagnosis was set, so it is not certain that all cases dying at a later age were also diagnosed at that stage. In the undiagnosed, the effect seemed to be greater in the elderly. Given that even the subclinical form of IHD has a tendency to progress over time, especially when left undiagnosed and untreated, this finding too is sensible. Many processes related to ageing, such as central arterial stiffness which amplifies the hypertensive response to cold exposure^19^, might in part explain these results.

There were differences in effect modification by sex between the diagnosed and the undiagnosed. Among the undiagnosed, the effect estimate of cold spells was greater among women. It is possible that IHD in women is more prone to be misdiagnosed as something else as men might manifest more classical symptoms^20^. Therefore some of these cases might have been sick without proper medication. Among the diagnosed, the effect estimate was greater for men than for women. One possible explanation is that in Northern Finland, the compliance to behavioral adjustments and pharmacological therapies might be poorer amongst men than amongst women. Therefore some of these cases, too, might have been sick without proper medication. However, there is no available local scientific evidence on the gender differences in compliance which is commonly witnessed at the clinics. These interpretations are therefore speculative.

There were differences in effect modification by prior diagnosis of IHD. The effect estimate was stronger for those without a previously diagnosed IHD compared to those who had been diagnosed during their lifetime. This finding makes sense not only in the context of our hypotheses, but also because SCD is known for commonly being the first clinical manifestation of the underlying cardiac disease or occurring among individuals who have been considered at low risk of cardiovascular death^3^. This characteristic of SCD does not only highlight its villainous nature and the importance of continuous efforts towards improved risk stratification^13^; It also highlights the importance of identifying previously unrecognized environmental triggers of SCD, such as cold spells, that could be avoided with relative ease in the broader context of public behavior^8^. We believe that more research efforts should be allocated to investigate the associations between weather and SCD.

**Figure 1:**
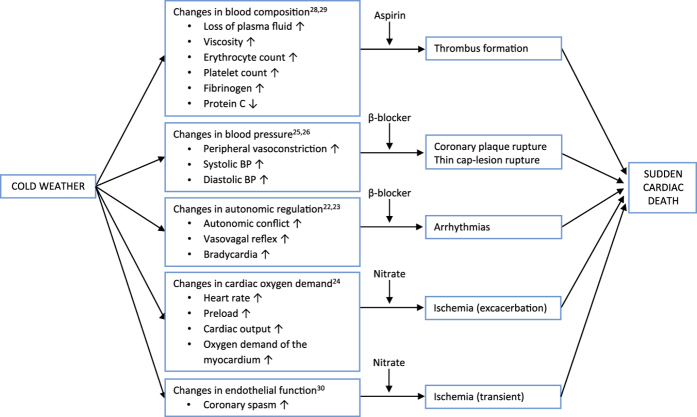
Illustration of the suggested pathophysiological pathways mediating the effects of cold spells on SCD. Potential effect modification by a cardioprotective medication is marked in each pathway. The pathways are discussed further in the main text, and the selected references have been limited to few examples.

The use of β-blocking medication seemed to decrease the effect of cold spells, regardless of whether the indication for β-blocker had been IHD or hypertension. The importance of β-blockers in both primary and secondary prevention of SCD has been established^21^, but this is, to our knowledge, the first time the role of β-blockers as potential modifiers of the harmful health effects of cold weather has been formally investigated. Our finding is consistent with our hypothesis and is pharmacologically and pathologically credible. Cold exposure activates both sympathetic and parasympathetic nervous systems, promoting an autonomic conflict which may potentially lead to arrhythmias (Fig. 1)^22^,^23^. The fact that β-blockers seem to provide protection towards the harmful effects of cold spells suggests that preventable arrhythmias play a role in some of the cases and should encourage physicians and patients to improve compliance of the use of β-blockers^21^. Cold exposure is also known to induce rapid increases in blood pressure^24^,^25^,^26^, which might increase the risk of coronary plaque rupture. The anti-hypertensive attributes of β-blockers attenuate this physiological response (Fig. 1).

The use of aspirin seemed to decrease the effect of cold spells among those with a prior diagnosis of IHD. Aspirin is well known to reduce serious vascular events and is commonly used for secondary prevention of myocardial infarction in patients with a high risk of developing an acute coronary event^27^. However, to our knowledge, this is one of the first times the role of aspirin as a potential modifier of the harmful effects of cold weather has been formally investigated. In a previous study conducted on populations from England and Wales^14^, aspirin was found to decrease the risk of acute myocardial infarction during cold weather. Our finding is consistent with our hypothesis and is pharmacologically and pathologically credible. Thrombogenic complications are associated with coronary plaques^3^, and cold weather increases the risk of thrombogenic complications by inducing well-known and relatively rapid changes in blood composition, such as haemoconcentration, higher concentrations of blood clotting factors, and lower concentrations of antithrombotic factors (Fig. 1)^28^,^29^. The fact that aspirin seems to provide protection towards the harmful effects of cold spells indicates that thrombogenic complications play a role in some of the cases of SCD. This should encourage physicians and patients to continue using aspirin for prevention of acute coronary events.

The use of nitrates seemed to decrease the effect of cold spells among those with a prior diagnosis of IHD. This is, to our knowledge, the first time the role of nitrates as potential modifiers of the harmful effects of cold weather has been formally investigated. Regardless of whether nitrates are known to improve the prognosis in patients with IHD, our finding in the context of cold weather is pharmacologically and pathologically credible and consistent with our hypothesis. Exposure to cold ambient temperature may induce coronary spasms in various groups of patients^30^, and both short- and long-acting nitrates may defuse the spasms, alleviate the symptoms, and perhaps even provide the patient with more time for contacting emergency medical services (Fig. 1). Exposure to cold air is also known to increase cardiac oxygen demand through various mechanisms (Fig. 1)^24^. Especially in patients with coronary atherosclerosis and significant stenosis, the earlier exhaustion of the vasodilator reserve combined with the attenuated endothelial-mediated vasodilatation in response to cold might lead to mismatch between myocardial oxygen demand and supply, resulting in ischemia^24^. Simply put, the additional strain during cold weather might compromise an already complicated situation in the severely ill.

The strengths of our study were the specific *a priori* hypotheses, and the strong study design to address these hypotheses. The hypotheses were based on prior epidemiological evidence on the relation between cold ambient temperature and cardiovascular mortality, and on prior experimental evidence on human cardiovascular and haematological responses to cold ambient temperatures. Some of these responses and pathways are illustrated in Fig. 1 together with the suggested pharmacological effect modifiers. The hypotheses were tested using the world’s largest autopsy-verified dataset on SCD. There is evidence that the diagnosis of SCD should be confirmed by medico-legal autopsy and histological sampling for validity^2^. It is difficult to distinguish between cardiac and non-cardiac cause of sudden death without an autopsy, since many conditions that evolve rapidly, such as aortic dissection, massive pulmonary embolism, or stroke, can lead to sudden collapse and death. Register based data and retrospective death certificate based methodology might overestimate the incidence of SCD by as much as 200 to 300%^2^.

Another strength of our study was the novel exposure assessment based on the concept of personal frequency distribution in the definition of exceptionally cold weather. Previously, it has been common practice to derive the (shared) frequency distribution from temperatures over the entire study period, which identifies unusually cold events mainly during the coldest winter months. Improvements to the definition of cold spell were introduced based on the findings of our recent systematic review and meta-analysis^9^. Together with the 51 years of weather data and the GIS based individual exposure assessment for the week preceding death, this increased the accuracy of the study on many levels.

A limitation of our study was that it was not possible to confirm whether the patients who had been prescribed the medications were actually taking them. However, if some of the cases were neglecting their prescribed medications, the resulting change in the effect estimates should be towards that of the non-users, not away from it. In this regard it seems plausible that the observed differences in the effect estimates between the users and non-users of medications represent real differences in relative risk.

Another limitation of our study was that we were unable to distinguish between different subtypes of medications, such as short- and long-acting nitrates or specific β-blockers. Even if such information would have been available, the resulting groups would likely have been too small for the regression model to converge. New approaches need to be developed in order to further elaborate these findings.

Another limitation of our study was that a total of 311, 310, and 281 cases were present with missing values for the prescriptions of aspirin, β-blockers, and nitrates, respectively. This is an unlikely source of bias because of the standardized process of initially gathering the information^17^,^31^. Nevertheless, as a precaution we excluded cases with missing values from each respective stratified analysis, which decreased the statistical power of the analyses.

Finally, we did not assess the potential effect modification of other existing medications or diagnoses of the cases. The selection of the analyzed pharmacologic agents was based on clear hypotheses and previous mechanistic physiological evidence related to cold exposure (Fig. 1), and we decided to restrain from explorative approaches in expanding this selection. The possibility of some other medical conditions influencing the effect of cold spells on the risk of SCD can’t be overruled, but we are not aware of any such agents which, in addition, should be unevenly distributed among the diagnosed and the undiagnosed to bias the estimates. All in all, our results form a coherent entity in which each effect estimate is manifesting the same direction of effect modification.

We conclude that cold spell is a potent determinant of SCD. Patients without a proper diagnosis of IHD seem more susceptible to the effects of cold spells on the risk of SCD than patients who are diagnosed during their lifetime. Aspirin, β-blockers, and nitrates seem to alleviate the effect of cold spells. Timely recognition of IHD and the pharmacotherapy aimed at the diagnosed seem meaningful ways of mitigating SCD during cold spells. These findings should be repeated, and the long-lost concept of weather as a determinant of SCD should be redeemed for the potential impacts on global public health. Further studies on the pharmacological modification of the harmful effects of extreme weather are likely to provide more insight to the pathological mechanisms and pathways which underlie the effects of weather on health.

## Methods

### Study Design

We conducted a case-crossover study to assess and elaborate the relation between cold spells and ischaemic SCD. A case-crossover design is well suited for studying the effects of transient short-term exposures on the risk of acute events^32^. We assigned each case a hazard period (HP) of 7 days preceding the day of death and 50 reference periods (RP) comprising of the same calendar days of the other years of the study period 1961–2011.

### Study Population

The study population included 3614 consecutive cases of SCD (1998–2011) from The Finnish Study of Genotype and Phenotype Characteristics of Sudden Cardiac Death (FinGesture) in the Province of Oulu, Northern Finland^17^,^31^. Ischaemic etiology was the underlying cause of death in 2572 cases aged ≥35. FinGesture is, to our knowledge, the largest existing study on autopsy-verified SCD in the world. All cases selected for this study had atherosclerotic heart disease (ICD-10 I25.1) as the underlying cause of death, defined by the autopsy finding. Case validation was conducted by a forensic specialist at the Department of Forensic Medicine of the University of Oulu through medicolegal autopsy, detailed histological sampling for all cases, full medical history, and police reports. Those with any other cause than SCD, including intoxications, were excluded. All witnessed SCDs occurred within 6 hours of the onset of the forewarning symptoms. All unwitnessed victims of SCD were seen alive and in a normal state of health within 24 hours preceding the death. Post-mortem studies of unexpected death are legally mandatory in Finland, and the selection bias in victims with unexpected SCD is minimal^17^. Hence, this provides a unique opportunity to cover the entire breadth of SCD cases in the general population.

For the purpose of assessing effect modification related to the setting of a proper diagnosis before death, prior IHD was defined as a prior diagnosis of CAD (ICD-10 I25) and/or AP (ICD-10 I20) and/or the survival of a previous AMI (ICD-10 I21). We refer to those with a prior diagnosis of IHD as “diagnosed”.

### Exposure Assessment

Geographical Information System (GIS) was used to allocate daily temperatures at the home coordinates of each case for the days in hazard and reference periods over the study years 1961–2011. For this purpose, a dataset containing minimum, mean, and maximum daily temperatures in the study period was obtained from the Finnish Meteorological Institute. In this dataset, point measurements have been interpolated onto a 10 × 10 km grid covering the whole of Finland, using a Kriging method^33^. We organized the temperature dataset into a GIS database, and GIS based functions were then used to extract temperature values from the database according to location and date.

We formed a personal frequency distribution of daily temperatures during the hazard and reference periods for the home coordinates of each case. Cold spell was defined as a period of ≥3 consecutive days with daily minimum temperature below the 5^th^ percentile of the personal frequency distribution. Conceptually this approach identifies events that are unusually cold in the respective place and time of the year. Personal exposure assessment was conducted for the hazard period. In contrast to this, the occurrence of cold spells during the reference periods represent the usual exposure at the individual’s place of residence. Thus, we estimated the occurrence of the weather phenomenon during the two period types and analyzed whether the possible differences in the occurrence could be explained by random variation.

### Statistical Methods

We applied conditional logistic regression to estimate the odds ratios (OR) and 95% confidence intervals (CI) representing the proportion of cold spells occurring during hazard and reference periods. Conditional logistic regression was performed using PROC PHREG in SAS (SAS, version 9.4, SAS Institute, Cary, NC) using the discrete logistic model and forming a stratum for each ID. We formed an indicator variable consisting of 5-year intervals over the study period to control for long time trends in the occurrence of cold spells. We conducted stratified analyses according to sex, age, prior diagnosis of IHD, and the use of aspirin, β-blockers, and/or nitrates.

### Ethical Considerations

This study complies with the Declaration of Helsinki and has been approved by the Ethics Committee of the Oulu University Hospital. National Supervisory Authority for Welfare and Health (VALVIRA) has approved the review of post-mortem data by the investigators.

## Additional Information

**How to cite this article**: Ryti, N. R. I. *et al*. Cold spells and ischaemic sudden cardiac death: effect modification by prior diagnosis of ischaemic heart disease and cardioprotective medication. *Sci. Rep.* **7**, 41060; doi: 10.1038/srep41060 (2017).

**Publisher's note:** Springer Nature remains neutral with regard to jurisdictional claims in published maps and institutional affiliations.

## Figures and Tables

**Table 1:** Characteristics of the study population.

	Diagnosed n (%)	Undiagnosed n (%)	Total n (%)
All	823 (32%)	1749 (68%)	2572 (100%)
Age			
Age 35–64	286 (35%)	844 (48%)	1130 (44%)
Age ≥ 65	537 (65%)	905 (52%)	1442 (56%)
Sex			
Men	677 (82%)	1383 (79%)	2060 (80%)
Women	146 (18%)	366 (21%)	512 (20%)

Absolute numbers and percentages of cases suffering ischaemic SCD. Diagnosed, the case had been diagnosed with ischaemic heart disease during lifetime.

**Table 2:** Distributions of temperature and exposure parameters at the home addresses of the cases.

Variable	Total
Average Temperature, mean (SD), °C	−2.36, 10.90
Temperature Range, °C	67.70
Lowest Temperature, °C	−45.80
Highest Temperature, °C	+ 21.90
Temperature Quartiles, Q1, Q2, Q3, °C	−8.70, −0.70, + 6.00
Individual Days Below Threshold, n (%)	42749 (0.04%)
Number of Identified Cold Spells, n	2817

Study years 1961–2011. The temperatures are expressed in degree Celsius and represent the mean of all daily values in the home coordinates of the cases for the periods of interest. SD, standard deviation; Q1, 25th percentile; Q2, 50th percentile, Q3, 75th percentile; Cold spell, ≥ 3 consecutive days below the personal threshold temperature.

**Table 3:** Relation between the occurrence of cold spells and the risk of ischaemic SCD according to age, sex, and prior diagnosis of ischaemic heart disease.

	Diagnosed OR (95%CI)	Undiagnosed OR (95%CI)	All OR (95% CI)
All	1.14 (0.61–2.10)	1.70 (1.13–2.56)	1.49 (1.06–2.09)
Age			
Age 35–64	2.45 (0.91–6.64)	1.42 (0.77–2.62)	1.63 (0.97–2.75)
Age 65+	0.77 (0.34–1.73)	1.99 (1.14–3.45)	1.39 (0.89–2.18)
Sex			
Men	1.23 (0.63–2.42)	1.56 (0.98–2.48)	1.44 (0.98–2.11)
Women	0.78 (0.17–3.63)	2.35 (0.98–5.66)	1.69 (0.80–3.56)

Unadjusted odds ratios (OR) and 95% confidence intervals (CI) representing the relation between the occurrence of cold spells and the risk of ischaemic SCD. Diagnosed, the case had been diagnosed with ischaemic heart disease during lifetime.

**Table 4:** Relation between the occurrence of cold spells and the risk of ischaemic SCD according to specific prior diagnosis.

	n (%)	OR (95% CI)
Coronary Artery Disease		
Yes	661 (26%)	1.12 (0.58–2.20)
No	1860 (74%)	1.74 (1.17–2.59)
Acute Myocardial Infarction		
Yes	264 (11%)	0.35 (0.08–1.52)
No	2246 (90%)	1.69 (1.18–2.43)
Angina Pectoris		
Yes	225 (9%)	1.21 (0.33–4.45)
No	2260 (91%)	1.50 (1.05–2.16)

Absolute numbers of cases, percentages per each diagnosis, and unadjusted odds ratios (OR) and 95% confidence intervals (CI) representing the relation between the occurrence of cold spells and the risk of ischaemic SCD. “Yes” indicates the patient had been diagnosed with the condition during lifetime, and “no” indicates the patient had not been diagnosed with the condition during lifetime.

**Table 5:** Relation between the occurrence of cold spells and the risk of ischaemic SCD according to use of medications.

	n (%)	OR (95% CI)
Use of Medications		
No medications	76 (15%)	4.23 (1.06–16.94)
Any medications	435 (85%)	1.05 (0.45–2.42)
All medications	113 (22%)	0.51 (0.06–4.22)
Aspirin		
No	285 (56%)	1.90 (0.77–4.70)
Yes	227 (44%)	1.02 (0.34–3.08)
β-blockers		
No	212 (41%)	1.61 (0.63–4.11)
Yes	301 (59%)	1.29 (0.46–3.60)
Nitrates		
No	192 (35%)	3.95 (1.52–10.25)
Yes	350 (65%)	0.52 (0.15–1.74)

Absolute numbers and percentages of the diagnosed cases using medications, and unadjusted odds ratios (OR) and 95% confidence intervals (CI) representing the relation between the occurrence of cold spells and the risk of ischaemic SCD. “No medications”, “any medications”, and “all medications” refer to aspirin, β-blockers, and nitrates. Cases with missing information per each medication have been excluded from the respective analyses.
